# Development of a Greek Oral health literacy measurement instrument: GROHL

**DOI:** 10.1186/s12903-020-1000-5

**Published:** 2020-01-15

**Authors:** Konstantina Taoufik, Kimon Divaris, Katerina Kavvadia, Haroula Koletsi-Kounari, Argy Polychronopoulou

**Affiliations:** 1 Department of Community and Preventive Dentistry, School of Dentistry, National and Kapodistrian University of Athens, Athens, Greece; 2 Department of Pediatric Dentistry, School of Dentistry, University of North Carolina-Chapel Hill, Chapel Hill, NC USA; 3 Department of Epidemiology, UNC Gillings School of Global Public Health, University of North Carolina-Chapel Hill, Chapel Hill, NC USA; 4 Department of Pediatric Dentistry, School of Dentistry, University of Louisville, Louisville, KY USA

**Keywords:** Oral health literacy, Literacy instrument, Oral health, Health literacy, Validity, REALD-30

## Abstract

**Background:**

Oral health literacy is an important construct for both clinical and public health outcomes research. The need to quantify and test OHL has led to the development of measurement instruments and has generated a substantial body of recent literature. A commonly used OHL instrument is REALD-30, a word recognition scale that has been adapted for use in several languages. The objective of this study was the development and testing of the Greek language oral health literacy measurement instrument (GROHL).

**Methods:**

Data from 282 adult patients of two private dental clinics in Athens, Greece were collected via in-person interviews. Forty-four words were initially considered and tested for inclusion. Item response theory analysis (IRT) and 2-parameter logistic models assessing difficulty and discriminatory ability were used to identify an optimal scale composition. Internal consistency was examined using Cronbach’s *alpha* and test-retest reliability was measured using intraclass correlation coefficient (ICC) in a subset of 20 participants over a two-week period. Convergent validity was tested against functional health literacy screening (HLS) items, dental knowledge (DK), oral health behaviors (OHBs), oral health-related quality of life (OHRQoL; OHIP-14 index), as well as self-reported oral and general health status.

**Results:**

From an initial item pool of 44 items that were carried forward to IRT, 12 were excluded due to no or little variance, 10 were excluded due to low item-test correlation, and 2 due to insignificant contribution to the scale, i.e., difficulty parameter estimate with *p* > 0.05. The twenty remaining items composed the final index which showed favorable internal consistency (*alpha* = 0.80) and test-retest reliability (ICC = 0.95). The summary score distribution did not depart from normality (*p* = 0.32; mean = 11.5; median = 12; range = 1–20). GROHL scores were positively correlated with favorable oral hygiene behaviors and dental attendance, as well as HLS, DK and education level.

**Conclusion:**

The GROHL demonstrated good psychometric properties and can be used for outcomes research in clinical and public health settings.

## Background

During the last decade, health literacy has emerged as a novel, important element of social and behavioral pathways underpinning oral health outcomes [1–3]. Specifically, health literacy in the oral health domain [oral health literacy, (OHL)] is understood as “the degree to which individuals have the capacity to obtain, process, and understand basic oral health information and services needed to make appropriate health decisions” [4, 5]. OHL encompasses a potentially modifiable yet complex set of knowledge and skills, distinct from general education—it is regarded as major determinant of one’s ability to make meaningful decisions regarding a wide spectrum of oral health-related behaviors and activities. Consequently, knowledge of OHL can explain patterns of suboptimal use of health services, dental care avoidance, foregoing preventive oral health behaviors and engaging in deleterious ones [6–10]. Furthermore, providers working with low health literacy individuals or populations, may need to accommodate and adjust their communication approaches and specific messages at an appropriate OHL level [11, 12].

The need to quantify and test OHL has led to the development of measurement instruments and has generated a substantial body of recent literature. Several instruments and scales have been introduced to measure OHL, including word recognition (e.g., REALD-99 and REALD-30) [13, 14], comprehension [15, 16] and functional skills-based tools (e.g., TOFHLID) [17]. These instruments have been used in a variety of settings and among diverse populations, internationally. The emanating body of literature has verified the postulated associations between OHL and a host of behaviors and outcomes, among adults [6–9] and their children [10, 18–20].

A recent systematic review of OHL measurement tools reported by Parthasarathy and colleagues [21] discusses the strengths and weaknesses of available measurement instruments. The authors suggest that additional work to refine and validate most tools is needed, and further research is warranted to measure broader conceptualizations of OHL, address cross-cultural and language adaptation issues. It was also noted that 23 of 29 OHL reviewed studies were conducted in the United States—European studies investigating OHL as an oral health determinant are generally scarce, in spite of almost half of Europeans are known to have inadequate or problematic health literacy skills [22]. The most commonly used OHL instrument is REALD-30—a word recognition scale that has been adapted for use in several languages other than English [23–27], but not Greek. In fact, currently no Greek language OHL measurement instrument exists. To address this gap, this study sought to develop and validate a Greek-language OHL instrument (GROHL).

## Methods

### Study population

Previous studies reporting the development of OHL instruments have used samples sizes in the range of 100–200 participants [14, 23–27]. Specifically, previous reports of the development of REALD-99 [13] and REALD-30 were based upon sample sizes of 102 and 202 adults [14], respectively. Based on this information, we did not conduct a formal sample size estimation or power calculation and sought to enroll a sample of over 200 individuals. Thus, we recruited a consecutive convenience sample of 300 adults who were seeking care at two private practice dental clinics in Athens, Greece. The choice of two private dental clinics as recruitment venues was motivated by an effort to capture individuals who were seeking mainstream dental care versus specialized care at an academic center. The inclusion criteria were self-reported ability to speak, understand and write in the Greek language. Exclusion criteria were inability to read, understand and speak Greek due to vision/hearing problems or any other reason, and working in areas associated with oral health. A second, independent sample of 20 adults was recruited, using the same inclusion and exclusion criteria, for the purposes of rest-retest reliability evaluation of the index, over a two-week period. Five of the 300 individuals that were sequentially screened for eligibility were determined to be ineligible. Of the remaining 295, 282 agreed to participate in the study—a response rate of 96%. The study received ethics approval from the institutional review board of the Athens University School of Dentistry and all participants provided a signed, informed consent.

### Data collection

To reduce biases associated with low literacy affecting data collection completeness and quality, data collection was done using an interview format. A structured questionnaire was used by a single investigator/interviewer (first author) to conduct all participant interviews. Each interview lasted approximately forty minutes. The collected data domains included socio-demographics, self-reported health status and behaviors, oral health knowledge, health/oral health literacy, and oral health-related quality of life (OHRQoL). Beyond this information and as part of the GROHL development, the initial pool of words tested for reading and recognition comprised an array of 44 words. The administration of the new, under development index was not timed separately and was not recorded; nevertheless, its maximum duration did not exceed 6 min.

Information on health status and health behaviors was obtained from questionnaire items regarding overall health and oral health, dental visits and oral hygiene practices. More specifically the participants were asked 2 self-rated health questions (possible answers: excellent, very good, good, fair, poor, very poor, I don’t know, prefer not to answer); when their last dental visit occurred and why; and what type of dental treatment they have received over the years. They were also asked how often they brush their teeth and use dental floss, interdental brushes and mouthwash. Oral health knowledge was measured using an array of 16 true or false statements which each participant was asked state their agreement or disagreement with (a ‘don’t know’ option was also possible). Correct answers were scored as 1 and incorrect or ‘don’t know’ were scored as 0. These items were employed in two recent studies investigating the association between oral health literacy and oral health-related knowledge [28]. Three additional items were used to evaluate health/oral health literacy. OHRQoL was measured with a Greek-language version of the Oral Health Impact Profile (OHIP-14) [29].

### Development and administration of the GROHL word inventory

The development of GROHL departed from an initial pool of 44 candidate words selected from the published and validated English version of the REALD-30 instrument [14] with their explanatory words (OHLA-E), as well as an additional 14 words chosen from the longer version of the instrument, the similarly validated REALD-99 [13]. In our selection of these additional 14 words, we excluded those that are used in daily routine without specific relevance to dentistry (e.g., diet, habits, snacking, approval), those having a stronger association with medicine or general health care than dentistry (e.g., cancer, diabetes), as well as very common words in the Greek language, even if they were related to dentistry (e.g., tongue, dentist). In sum, we sought to be maximally inclusive of initial pool items that could serve the scale purpose, while excluding items that we determined upfront that would not perform well. All these words were strongly associated with oral health, and were agreed upon by consensus of two dental professionals/investigators—thus, demonstrating face validity. Content validity was not explicitly tested in this Greek-language adaptation of the instrument; however, its English-language counterparts have been the most extensively used OHL instrument used in the literature. Criterion validity was determined upon the examination of GROHL’s correlation with oral health behaviors and knowledge, oral health knowledge, health literacy screeners, and OHRQoL. Reliability was determined via Cronbach’s *alpha*.

Translation of the initial 44 words in the item pool was done using English-Greek dictionaries using professional knowledge/expertise when needed. The final translated version was further reviewed by two dental academicians and investigators who were proficient in English and produced a back-translation of the initial Greek instrument into English. This final version was also screened and verified in terms of language and with an independent native speaker and translator. All REALD-99 and REALD-30 items that were used as the initial pool of words to be tested, their recognition-test accompanying words, and their Greek-language counterparts, are presented in the Supplemental Table (Additional file 1: Table S1). Briefly, reasons for exclusion in the construction of the GROHL-20 included: no or little (< 5%) variance (*n* = 12) in responses, item-test correlation of less than 0.40 (*n* = 10) and test difficulty parameter estimate with *p* > 0.05 (*n* = 2).

Each participant was given a laminated copy of the 44 oral health-related Greek words list that comprised the initial GROHL pool of candidates. Participants were asked to read aloud each word and state whether they knew what the word meant—they were instructed not to guess. For the words that were positively identified, a follow-up comprehension quiz was given: participants were asked to pick one of two words that most closely resembled the index word. For instance, “sugar”: sweet or sour. Finally, they were asked to explain the meaning of the main word and the investigator assessed whether the participant understood the meaning of the word, based on a definition checklist created from a reference dictionary. Pronunciation and recognition were scored for each word. From a methodological standpoint, if a participant hesitated or read the word slowly, s/he would be reminded that one should only read the words associated with dentistry that s/he knows the meaning of and not guess. If a participant was positive that s/he knew the word, this would be considered as ‘correct’ and we would proceed to the comprehension quiz. Of note, no such ambiguous events took place during our study, likely owing to the phonetic nature (i.e., there is a direct correlation between the spelling and the sound of each word) of the Greek language. Of note, the scoring of both pronunciation and recognition resembles closely the Spanish OHL instrument development [15] compared to the English version [14], which is only based upon pronunciation.

### Analytical approach

Initial data analysis relied upon descriptive statistics (e.g., frequencies, proportions, means, standard deviations, medians, ranges), and bivariate analyses (e.g., Student’s *t* test, ANOVA), reported using tabular and visual means. Item response theory analysis and 2-parameter logistic models assessing variance, difficulty, discriminatory ability and item-test correlations were used to identify an optimal scale composition. The scale’s test-retest reliability among twenty individuals over a two–week period was measured using the intraclass correlation coefficient (ICC). Internal consistency was measured using Cronbach’s *alpha*. Convergent validity was tested against functional health literacy screening (HLS) items, dental knowledge (DK), oral health behaviors (OHBs), OHRQoL (OHIP-14 index), as well as self-reported oral and general health status. Composite scores were computed and used for health literacy screeners (3 items; score ranging between 3 and 12; alpha = 0.65) and dental knowledge (16 items; score ranging between 0 and 16; alpha = 0.59). Spearman (*rho*) rank correlations between GROHL scores and other constructs or variables of interest were obtained. *P*-values are presented rounded to one significant digit [30]. P-values were not corrected for multiple testing and values less than 0.05 were considered significant. All analyses were done using Stata 16.0 (StataCorp LP, College Station, TX).

## Results

**Fig. 1:**
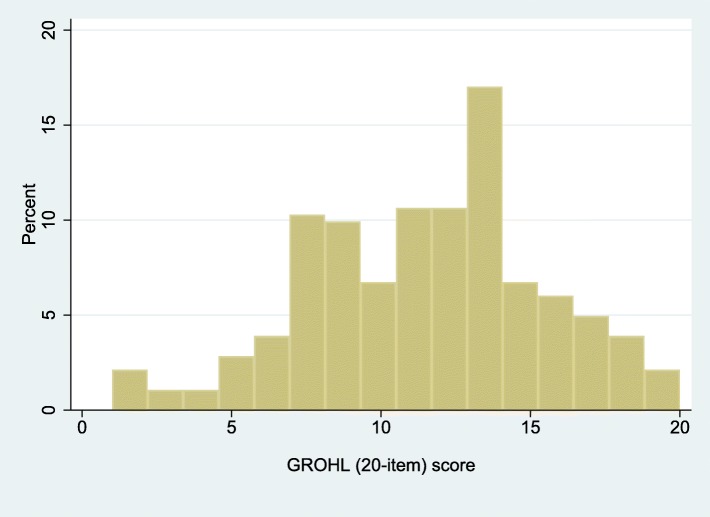
Distribution of the Greek Oral Health Literacy Measurement Instrument (GROHL) scores

**Fig. 2:**
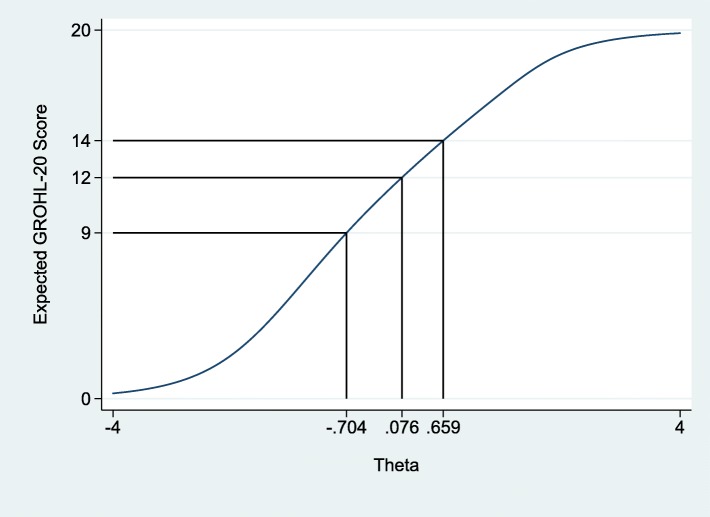
Test characteristic curve of the Greek Oral Health Literacy Scale, with *theta* values corresponding to the quartile limits of the index score distribution (9, 12 and 14) among the 282 study participants

**Fig. 3:**
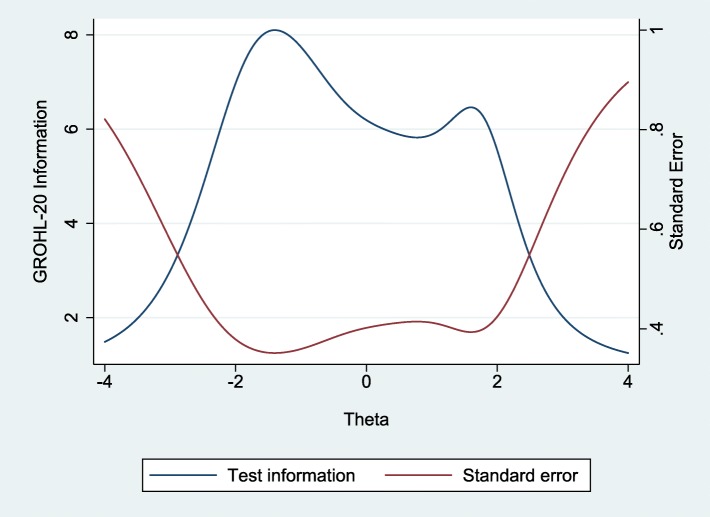
Test information and standard error functions of the Greek Oral Health Literacy Scale across *theta* values among the 282 study participants

The final list of items included in GROHL-20 is presented in Table 1 and the demographic information of the 282 participating individuals is presented in Table 2. Briefly, the majority of participants, were women, married, with technical or university education, and were of mean age 39 years. Using these individuals’ responses and departing from the initial 44 words, we first excluded 12 words due to no or insufficient invariance. Ten additional words were removed due to low (< 0.40) item-test correlation and two more for non-significant contribution (i.e., difficulty estimate > 0.05) to the scale (Additional file 1: Table S1). The remaining 20 words comprised the GROHL index. The GROHL’s summary score distribution (Fig. 1) did not depart from normality (D’Agostino skewness and kurtosis test: *p* = 0.32; mean = 11.5; standard deviation = 4.0; median = 12; range = 1–20). The scale showed good internal consistency (*alpha* = 0.80) and excellent test-retest reliability (average ICC = 0.95; *p* < 0.0005). Overall, the GROHL score showed favorable distribution with most information and discriminatory potential demonstrated around the center and towards the low-end of the literacy construct (represented as *theta*; Figs. 2 and 3).

**Table 1:** GROHL-20 Item information and test scale estimates

Item no.	English language REALD item	Greek language GROHL-20 item	Discriminatory ability; coefficient (standard error)	Difficulty; coefficient (standard error)	Item-test correlation	Item-rest correlation	Average interim covariance	alpha
11	Genetics	Γενετική	1.73 (0.38)	−1.86 (0.26)	0.41	0.34	0.03	0.82
14	Enamel	Αδαμαντίνη	1.84 (0.33)	−1.16 (0.33)	0.54	0.47	0.03	0.81
15	Dentition	Οδοντοφυΐα	1.80 (0.37)	−1.66 (0.22)	0.43	0.39	0.03	0.82
17	Pulp	Πολφός	1.44 (0.26)	1.05 (0.17)	0.52	0.43	0.03	0.82
18	Malocclusion	Ανωμαλία Σύγκλεισης	1.15 (0.20)	− 0.45 (0.14)	0.50	0.40	0.03	0.82
20	Sealant	Κάλυψη Οπών και Σχισμών	0.96 (0.19)	−0.96 (0.21)	0.45	0.35	0.03	0.82
21	Periodontal	Περιοδοντικό	1.18 (0.23)	−1.41 (0.24)	0.44	0.35	0.03	0.82
22	Analgesia	Αναλγησία	1.89 (0.35)	−1.13 (0.21)	0.54	0.46	0.03	0.81
23	Fistula	Συρίγγιο	1.13 (0.21)	−0.84 (0.17)	0.50	0.40	0.03	0.82
24	Hyperemia	Υπεραιμία	1.69 (0.32)	−1.32 (0.18)	0.50	0.43	0.03	0.82
26	Bruxism	Βρυγμός	1.85 (0.38)	1.54 (0.20)	0.45	0.38	0.03	0.82
29	Temporomandibular	Κροταφογναθική	1.84 (0.30)	0.44 (0.11)	0.60	0.51	0.03	0.81
30	Apicoectomy	Ακρορριζεκτομή	1.20 (0.23)	1.29 (0.22)	0.44	0.35	0.03	0.82
31	Filling	Έμφραξη	1.64 (0.26)	0.23 (0.11)	0.60	0.51	0.03	0.81
32	Cavity	Κοιλότητα	2.36 (0.58)	−1.90 (0.24)	0.42	0.37	0.03	0.82
34	Eruption	Ανατολή	1.20 (0.22)	1.06 (0.19)	0.47	0.37	0.03	0.82
40	Incisor	Τομέας	0.95 (0.18)	0.83 (0.20)	0.44	0.34	0.03	0.82
41	Splint	Ακινητοποίηση	1.33 (0.24)	−0.94 (0.16)	0.52	0.43	0.03	0.82
42	Mouthguard	Νάρθηκας	1.04 (0.21)	−1.25 (0.23)	0.45	0.36	0.03	0.82
43	Avulsion	Εκγόμφωση	3.12 (0.93)	1.77 (0.20)	0.40	0.35	0.03	0.82
Test scale							0.03	0.83

**Table 2:** Sociodemographic characteristic and oral health literacy estimates of the 282 study participants

			GROHL	
	n or mean	% or SD	mean (SD)	P^*^
Entire sample	282	100	11.5 (4.0)	
Sex				0.8
female	251	89	11.4 (4.0)	
male	31	11	11.6 (4.0)	
Education (categorical)				< 0.0005
high school or less	51	18	8.6 (4.0)	
technical college	94	33	11.2 (3.7)	
university	81	29	12.5 (3.7)	
graduate	56	20	13.0 (3.8)	
*years (mean, SD)*	15	2.8		
Age (years; quartiles)				0.5
*Q1* range: 23.5, 35.7	33	2.8	11.0 (3.7)	
*Q2* range: 35.7, 38.7	37	0.8	11.3 (3.8)	
*Q3* range: 38.8, 43.1	41	1.3	11.6 (4.0)	
*Q4* range: 43.2, 69.8	48	6.5	11.9 (4.4)	
*years (mean, SD)*	39	6.9		
Marital				0.2
single	18	6	11.6 (3.9)	
married	246	87	11.3 (4.0)	
divorced, separated, widowed	18	6	13.2 (3.6)	
Number of children				0.6
0	19	7	12.2 (4.0)	
1	79	28	11.5 (3.3)	
2	154	55	11.5 (4.1)	
3 or more	30	11	10.7 (4.6)	
Foreign-born				0.8
no	271	96	11.4 (4.0)	
yes	11	4	11.8 (3.7)	
Primary language				0.9
Greek	279	99	11.5 (4.0)	
other	3	1	11.7 (5.5)	

*SD* Standard deviation

*derived from analyses of variance (ANOVA)

**Fig. 4:**
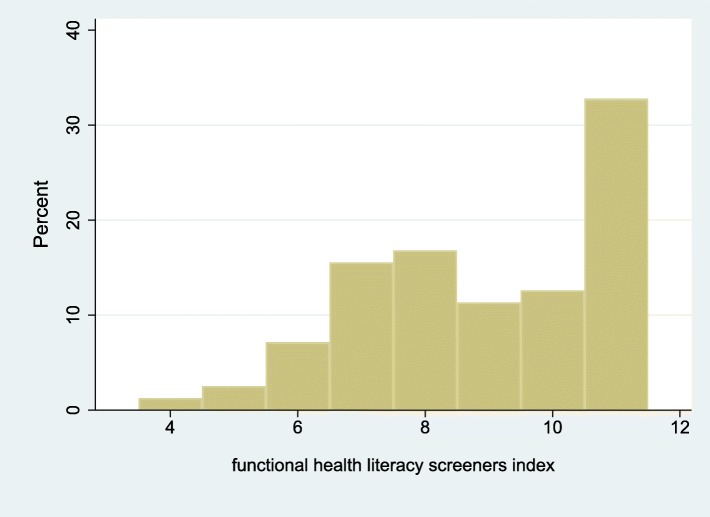
Distribution of the Functional Health Literacy index scores among the 282 study participants

**Fig. 5:**
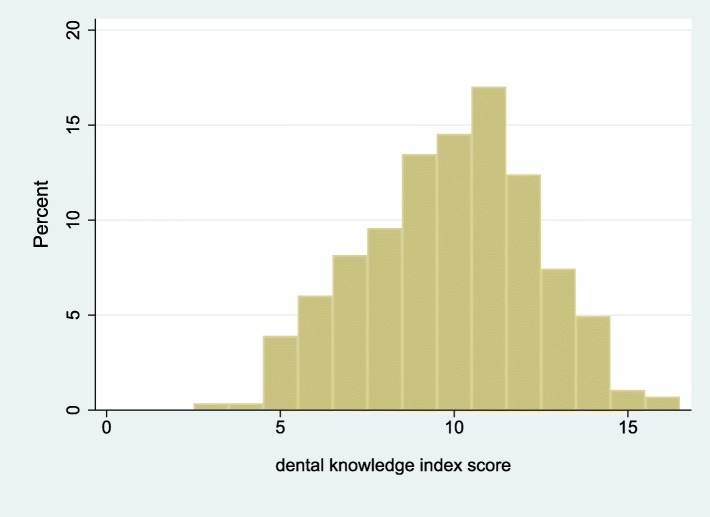
Distribution of the Dental Knowledge Index scores among the 282 study participants

GROHL scores were strongly positively associated with education (Table 2), recent and more frequent routine dental visits (Table 3), as well as use of mouthwash (Table 4). Smaller differences were noted with regard to tooth brushing frequency and use of dental floss. Higher GROHL scores were associated with better health literacy screening item responses and their composite score (Fig. 4); for instance, needing help to read health information material in a hospital and understanding written oral health information (Table 5). We didn’t note any important association between OHRQoL measures (i.e., prevalence, “extent” and “severity” of impacts; Table 6). However, we found a significant positive association (*rho* = 0.30; *p* < 0.0005; Table 7) of GROHL with dental knowledge scores (Fig. 5).

**Table 3:** General and oral health status and care-seeking attitudes and their associations with oral health literacy

SD, standard deviation			GROHL	
	n	col. %	mean (SD)	P^*^
Entire sample	282	100	11.5 (4.0)	
General health status				0.3
Excellent	31	11	12.3 (4.3)	
Very good	140	50	11.3 (3.7)	
Good	85	30	11.2 (4.1)	
Fair-poor	25	9	12.5 (4.3)	
Oral health status				0.6
Excellent	8	3	12.0 (3.5)	
Very good	60	21	12.0 (3.4)	
Good	126	45	11.2 (4.0)	
Fair-poor	88	31	11.4 (4.3)	
Last dental visit timing				0.05
< 12 months ago	186	66	11.8 (3.7)	
12–23 months ago	63	22	11.5 (4.2)	
2–5 years ago	22	8	9.6 (4.1)	
> 5 years go	6	2	9.7 (5.3)	
*don’t remember*	5	2		
Last dental visit reason				0.8
routine	168	60	11.5 (3.9)	
restorative	64	23	11.2 (4.0)	
problem/pain	49	17	11.5 (4.2)	
*don’t remember*	1	0		
Frequency of dental visits				0.05
every 6 months	74	26	12.4 (3.7)	
every year	118	42	11.6 (3.6)	
every 1–2 years	40	14	11.0 (4.1)	
Only when there is a problem	45	16	10.5 (4.6)	
*don’t remember*	5	2		

*derived from analyses of variance (ANOVA)

**Table 4:** Oral hygiene-related behaviors and oral health literacy estimates among the 282 study participants

			GROHL	
	n	col. %	mean (SD)	P^*^
Entire sample	282	100	11.5 (4.0)	
Daily tooth brushing frequency				0.2
twice or more	174	62	11.8 (3.9)	
once	102	36	11.0 (4.0)	
less than once	6	2	9.8 (5.3)	
Use of dental floss				0.3
yes	134	48	11.7 (3.8)	
no	147	52	11.2 (4.1)	
Use of mouthwash				0.001
yes	104	37	12.4 (3.6)	
no	177	63	10.8 (4.1)	

*SD* Standard deviation

*derived from analyses of variance (ANOVA) or Student’s *t* test

**Table 5:** Responses to functional health literacy screening items and composite health literacy screening (HLS) score distribution

			GROHL	
	n	col. %	mean (SD)	P^*^
I need help to read health information material when in a hospital				0.03
always	14	5	10.5 (4.3)	
sometimes	54	19	10.7 (4.4)	
rarely	55	19	10.9 (3.1)	
never	126	45	12.2 (4.0)	
*does not apply*	*33*	*12*		
I am confident in completing health forms				0.2
not at all	10	4	9.4 (3.2)	
somewhat	89	32	11.3 (4.1)	
very	170	60	11.7 (3.9)	
*does not apply*	*13*	*5*		
I have difficulty understanding written oral health information				0.001
always	9	3	10.7 (3.4)	
sometimes	66	23	10.0 (4.1)	
rarely	69	24	12.0 (3.4)	
never	122	43	12.2 (4.0)	
*does not apply*	*16*	*6*		
Composite HLS score				
*Median (range)*	9	4–11		
*Mean (SD)*	8.9	2.9		0.01
*“low” (< 7)*	26	11	10.1 (4.4)	0.05
*“adequate” (≥7)*	212	89	11.7 (3.9)	

*SD* Standard deviation

*derived from analyses of variance (ANOVA)

**Table 6:** Oral health-related quality of life estimates (OHIP-14 index) in the study sample

			GROHL	
	n	col. %	mean (SD)	P*
OHIP-14 prevalence of impacts				1.0
any impact	48	17	11.6 (3.9)	
no impact	228	83	11.6 (3.9)	
OHIP-14 “extent” of impacts				0.3
0	228	83	11.6 (3.9)	
1	22	8	10.7 (3.6)	
2	6	2	11.7 (2.8)	
3	6	2	10.7 (5.2)	
*4*	7	3	14.6 (3.8)	
5–11	7	3	12.1 (4.2)	
OHIP-14 “severity” of impacts				
mean (SD)	5	7.6		
median (range)	2	0–36		

*SD* Standard deviation

*derived from Student’s *t* test or analysis of variance (ANOVA)

**Table 7:** Correlations of oral health literacy with dental knowledge, OHRQoL, functional health literacy screener, and education

	Spearman’s *rho*	P
Dental knowledge index	0.30	< 0.0005
Functional health literacy index	0.21	0.001
OHRQoL OHIP-14 severity index	0.10	0.11
Education (years)	0.37	< 0.0005
Age (years)	0.12	0.04
Number of children	−0.06	0.34

## Discussion

Here we report the development and psychometric properties of a Greek-language OHL measurement instrument (GROHL). The scale development and testing were done among nearly 300 adults and followed common procedures and practices. Overall, we found that the GROHL demonstrated favorable psychometric properties and we recommend its further application and evaluation in additional populations, in clinical and public health settings. Additional properties of interest that can be studied in the future include, among others, its responsiveness to change (i.e., after educational interventions), its association with oral health care-seeking (i.e., our sample was limited to dental care-seeking individuals), and its potential for further item reduction (i.e., the development of a short-form GROHL version).

The scale’s development departed from its well-established English-language counterparts (REALD-30 and REALD-99) and it includes an additional, comprehension component that enables a double-scoring method, based upon both word pronunciation and recognition. GROHL scores were normally distributed and were positively correlated with a wide array of variables and constructs, including education, dental knowledge and oral hygiene behaviors. Although comparisons with literacy estimates from other studies and populations cannot be directly made, the mean score of 12/20 in this dental clinic-recruited population is within the low-end of the theorized range, in comparison to scores of 16/30 in a community setting [14], 21/30 in a University clinic [28] and 24/30 in a private dental clinic setting in the U.S. [31]. Based upon the score and information distribution of index score in this sample, a preliminary expectation would be that GROHL scores below 9 may indicate ‘insufficient’ OHL; this arbitrary threshold will certainly need to be empirically verified and interpreted separately in each application context. Of note, the index also demonstrated good internal consistency, similar to other non-English language adaptations of REALD-30.

The development of GROHL was done among a comparatively large sample of almost 300 dental care-seeking adults using rigorous item response theory-based criteria and subsequent psychometric evaluation. In fact, we used elements from both IRT and classical test theory (CTT), in a complementary manner [32]. For instance, we used IRT to determine the optimal set of items that we could carry forward and retain in our index based on their individual performance in terms of contributing information to the overall test score. Moreover, we conducted more ‘classical’ tests of scale reliability (i.e., test-retest, internal consistency, etc.) and overall performance. These are routine in the psychometric evaluation of new scales and add to our understanding of the performance of GROHL. As expected, several words used in the REALD family of indices were not included in the GROHL-20 based on the IRT analysis; these 24 words did not add to the information content of the index for various reasons, most likely due to the differential meaning and pronunciation between the English and Greek languages. In other words, our iterative IRT approach resulted in a high-information content set of items, that contribute to the differentiation of test takers across a wide spectrum of OHL. The double-scoring method employed, similar to the Spanish-version of REALD-30 (OHLA) [15] accounts for both pronunciation and recognition, which is, in our opinion, essential for the Greek language context. Of note, alternative scoring possibilities for GROHL exist (i.e., giving partial credit for pronunciation if recognition or comprehension criteria are not met, construct composite or weighted scores, etc.)—these schemes were outside the scope of the work presented here, but are promising future research directions for possible refinement of the index.

We found that GROHL scores were significantly positively correlated with overall educational attainment, dental-specific knowledge, oral health behaviors and attendance, as well as health literacy screening items. These findings should be interpreted with caution because dental-specific knowledge items and health literacy screeners, although used in earlier reports [4, 10, 29] were not validated or adapted in the context of our study. Nevertheless, these findings are aligned with earlier reports [6, 7, 13, 14] and corroborate the validity of the index. The fact that we did not find an association with OHRQoL does not imply that one does not really exist—it is conceivable that a “U-shaped” association exists, wherein individuals at the lowest and the highest ends of health literacy both over-report quality of life impacts, each for different reasons; the former due to poor oral health and the latter due heightened awareness, or elevated standards and expectations. Some evidence exists suggesting under-reporting of OHRQoL impacts (by caregivers for their children) associated with low oral health literacy [18].

As is the case for all developmental investigations of this nature, some limitations exist. First, the sampled population was actively oral health care-seeking and in fact at a dental clinic. This limits the potential of the sample to represent the community-dwelling population, as arguably those who are already at a dental office may be systematically different (in many ways, including in terms of health literacy) compared to their non-care-seeking counterparts. Further, word recognition and pronunciation are used here as proxies of functional health literacy, which pertains to actual skills (i.e., interpreting a prescription, recognizing signs of dental disease, following instructions to use a dental product or device, or performing oral hygiene tasks). These were not directly tested in this investigation. Arguably, tests of functional literacy are in principle superior because they assess actions or tasks of relevance to the domain of interest. For instance, the Hong Kong Oral Health Literacy Assessment Task for Pediatric Dentistry (HKOHLAT-P) [33] and the Test of Functional Health Literacy in Dentistry (TOFHLiD) [17] are based upon assessments of applied, oral health-related tasks and abilities, while word recognition and comprehensions tests serve as proxies of these task-performing abilities. The development of a functional (oral) health literacy instrument in the Greek language would be a welcome and likely necessary addition. In spite of this limitation, we support that the development and introduction GROHL is a step in the right direction--GROHL has favorable psychometric properties and was found to be significantly and positively associated with important health literacy screening questions that are demonstrative of daily, applied, healthcare-related skills. In sum, we foresee that the index will enable valid measurements of OHL, for the first time, in the Greek language.

## Conclusions

The introduction of this new Greek language oral health literacy index fills a gap in the toolbox available for oral health outcomes research. This is especially important in the domain of oral health literacy, as the construct is rapidly gaining traction and relevance in health care worldwide. Based on the results of this study, we support that the GROHL has good psychometric properties and can be used for outcomes research in clinical and public health settings. Further testing among non-clinical (i.e., not actively care-seeking) populations, as well as those living in rural areas will illuminate the performance of the index among diverse populations and settings.

## Supplementary information

**Additional file 1:** **Table S1.** presenting REALD-99, REALD-30, OHLA-E, GROHL pool, and GROHL-20 items in English and Greek

## Data Availability

The data used to generate and support the findings of this study are available from the corresponding author upon request.

## Abbreviations

DK: Dental knowledge
GROHL: Greek language oral health literacy instrument
HLS: Functional health literacy screening
ICC: Intraclass correlation coefficient
OHBs: Oral health behaviors
OHIP: Oral Health Impact Profile
OHLA-E: Oral Health Literacy Assessment with Explanatory words
OHRQoL: Oral health-related quality of life
REALD: Rapid Estimate of Adult Literacy in Dentistry
TOFHLID: Test of Functional Health Literacy in Dentistry

## References

[1] Nutbeam D (2000). Health literacy as a public health goal: a challenge for contemporary health education and communication strategies into the 21st century. Health Promot Int.

[2] DeWalt DA, Berkman ND, Sheridan S, Lohr KN, Pignone MP (2004). Literacy and health outcomes. J Gen Intern Med.

[3] Nutbeam D (2008). The evolving concept of health literacy. Soc Sci Med.

[4] Horowitz AM, Kleinman DV (2008). Oral health literacy: the new imperative to better oral health. Dent Clin N Am.

[5] Parker RM, Hernandez LM (2012). Oral health literacy: a workshop. J Health Commun.

[6] Lee JY, Divaris K, Baker AD, Rozier RG, Vann WF (2012). The relationship of oral health literacy and self-efficacy with oral health status and dental neglect. Am J Public Health.

[7] Lee JY, Divaris K, Baker AD, Rozier RG, Lee SY, Vann WF (2011). Oral health literacy levels among a low-income WIC population. J Public Health Dent.

[8] Parker EJ, Misan G, Chong A, Mills H, Roberts-Thomson K, Horowitz AM, Jamieson LM (2012). An oral health literacy intervention for indigenous adults in a rural setting in Australia. BMC Public Health.

[9] Firmino RT, Ferreira FM, Paiva SM, Granville-Garcia AF, Fraiz FC, Martins CC (2017). Oral health literacy and associated oral conditions: a systematic review. J Am Dent Assoc.

[10] Vann WF, Lee JY, Baker D, Divaris K (2010). Oral health literacy among female caregivers: impact on oral health outcomes in early childhood. J Dent Res.

[11] Rudd RE (2012). Oral health literacy: correcting the mismatch. J Public Health Dent.

[12] Horowitz AM, Maybury C, Kleinman DV, Radice SD, Wang MQ, Child W, Rudd RE (2014). Health literacy environmental scans of community-based dental clinics in Maryland. Am J Public Health.

[13] Richman JA, Lee JY, Rozier RG, Gong DA, Pahel BT, Vann WF (2007). Evaluation of a word recognition instrument to test health literacy in dentistry: the REALD-99. J Public Health Dent.

[14] Lee JY, Rozier RG, Lee SY, Bender D, Ruiz RE (2007). Development of a word recognition instrument to test health literacy in dentistry: the REALD-30–a brief communication. J Public Health Dent.

[15] Lee J, Stucky B, Rozier G, Lee SY, Zeldin LP (2013). Oral health literacy assessment: development of an oral health literacy instrument for Spanish speakers. J Public Health Dent.

[16] Villanueva Vilchis MD, Wintergerst A, Borges Yáñez SA (2015). Toward a comprehensive instrument of oral health literacy in Spanish. J Health Commun.

[17] Gong DA, Lee JY, Rozier RG, Pahel BT, Richman JA, Vann WF (2007). Development and testing of the test of functional health literacy in dentistry (TOFHLiD). J Public Health Dent.

[18] Divaris K, Lee JY, Baker AD, Vann WF (2012). Caregivers’ oral health literacy and their young children's oral health-related quality-of-life. Acta Odontol Scand.

[19] Divaris K, Lee JY, Baker AD, Gizlice Z, Rozier RG, DeWalt DA, Vann WF (2014). Influence of caregivers and children’s entry into the dental care system. Pediatrics.

[20] Vann WF, Divaris K, Gizlice Z, Baker AD, Lee JY (2013). Caregivers’ health literacy and their young children’s oral-health–related expenditures. J Dent Res.

[21] Parthasarathy DS, McGrath CP, Bridges SM, Wong HM, Yiu CK, Au TK (2014). Efficacy of instruments measuring oral health literacy: a systematic review. Oral Health Prev Dent.

[22] Hernandez LM, editor. Health literacy: Improving health, health systems, and health policy around the world: Workshop summary. National Academies Press; 2013. 24872976

[23] Stein L, Pettersen KS, Bergdahl M, Bergdahl J (2015). Development and validation of an instrument to assess oral health literacy in Norwegian adult dental patients. Acta Odontol Scand.

[24] Wong HM, Bridges SM, Yiu CK, McGrath CP, Au TK, Parthasarathy DS (2012). Development and validation of Hong Kong rapid estimate of adult literacy in dentistry. J Investig Clin Dent.

[25] Junkes MC, Fraiz FC, Sardenberg F, Lee JY, Paiva SM, Ferreira FM (2015). Validity and reliability of the Brazilian version of the rapid estimate of adult literacy in dentistry–BREALD-30. PLoS One.

[26] Tadakamadla SK, Quadri MF, Pakpour AH, Zailai AM, Sayed ME, Mashyakhy M, Inamdar AS, Tadakamadla J (2014). Reliability and validity of Arabic rapid estimate of adult literacy in dentistry (AREALD-30) in Saudi Arabia. BMC Oral Health.

[27] Peker K, Köse TE, Güray B, Uysal Ö, Erdem TL (2017). Reliability and validity of the Turkish version of the rapid estimate of adult literacy in dentistry (TREALD-30). Acta Odontol Scand.

[28] Miller E, Lee JY, DeWalt DA, Vann WF (2010). Impact of caregiver literacy on children's oral health outcomes. Pediatrics..

[29] Papagiannopoulou V, Oulis CJ, Papaioannou W, Antonogeorgos G, Yfantopoulos J (2012). Validation of a Greek version of the oral health impact profile (OHIP-14) for use among adults. Health Qual Life Outcomes.

[30] Altman DG, Bland JM (1996). Presentation of numerical data. BMJ..

[31] Jones M, Lee JY, Rozier RG (2007). Oral health literacy among adult patients seeking dental care. J Am Dent Assoc.

[32] Bechger TM, Maris G, Verstralen HH, Béguin AA (2003). Using classical test theory in combination with item response theory. Appl Psychol Meas.

[33] Wong HM, Bridges SM, Yiu CK, McGrath CP, Au TK, Parthasarathy DS (2013). Validation of the Hong Kong Oral health literacy assessment task for paediatric dentistry (HKOHLAT-P). Int J Paediatr Dent.

