# Supplementary material for: Development of a Greek Oral health literacy measurement instrument: GROHL
Source: BMC Oral Health. 2020 Jan 15;20:14. doi: 10.1186/s12903-020-1000-5 (PMC6964004; doi:10.1186/s12903-020-1000-5)
Supplement: Supplementary file 1 — Additional file 1: Table S1. presenting REALD-99, REALD-30, OHLA-E, GROHL pool, and GROHL-20 items in English and Greek [file 12903_2020_1000_MOESM1_ESM.docx]

|  | REALD-99  item | REALD-30  item | OHLA-E comprehension test words | | GROHL 44-item development pool | GROHL 44-item development pool comprehension test words | | Included in GROHL-20 final items list | Exclusion reason |
| --- | --- | --- | --- | --- | --- | --- | --- | --- | --- |
| 1 | Sugar | Sugar | Sweet | Bitter | Ζάχαρη | Γλυκό | Πικρό | NO | Non-variant |
| 2 | Smoking | Smoking | Lung | Stomach | Κάπνισμα | Πνεύμονας | Στομάχι | NO | Non-variant |
| 3 | Brush | Brush | Toothpaste | Soap | Βούρτσισμα | Οδοντόκρεμα | Σαπούνι | NO | Non-variant |
| 4 | Floss | Floss | Clean | Rinse | Νήμα | Καθαρίζω | Ξεπλένω | NO | Variance <5% |
| 5 | Gingiva | Gingiva | Pink | White | Ούλα | Ροζ | Άσπρο | NO | Non-variant |
| 6 | Fluoride | Fluoride | Protect | Destroy | Φθόριο | Προστατεύει | Καταστρέφει | NO | ITC <0.40 |
| 7 | Extraction | Extraction | Remove | Replace | Εξαγωγή | Αφαιρώ | Αντικαθιστώ | NO | Non-variant |
| 8 | Denture | Denture | Natural | Synthetic | Οδοντοστοιχία | Φυσικό | Τεχνητό | NO | Variance <5% |
| 9 | Incipient | Incipient | Early | Late | Αρχόμενο | Νωρίς | Αργά | NO | ITC <0.40 |
| 10 | Halitosis | Halitosis | Breath | Cough | Κακοσμία | Αναπνοή | Βήχας | NO | Non-variant |
| 11 | Genetics | Genetics | Friend | Family | Γενετική | Φίλος | Οικογένεια | **YES** |  |
| 12 | Restoration | Restoration | Instrument | Treatment | Αποκατάσταση | Εργαλείο | Θεραπεία | NO | ITC <0.40 |
| 13 | Braces | Braces | Straighten | Twist | Ορθοδοντικά αγκύλια | Ισιώνει | Παραμορφώνει | NO | ITC <0.40 |
| 14 | Enamel | Enamel | Surface | Inside | Αδαμαντίνη | Επιφανειακό | Εσωτερικό | **YES** |  |
| 15 | Dentition | Dentition | Mouth | Teeth | Οδοντοφυΐα | Στόμα | Δόντια | **YES** |  |
| 16 | Calculus | Calculus | Soft | Hard | Τρυγία | Μαλακό | Σκληρό | NO | ITC <0.40 |
| 17 | Pulp | Pulp | Tongue | Nerve | Πολφός | Γλώσσα | Νεύρο | **YES** |  |
| 18 | Malocclusion | Malocclusion | Bite | Wound | Ανωμαλία Σύγκλεισης | Δάγκωμα | Πληγή | **YES** |  |
| 19 | Caries | Caries | Ulcer | Cavity | Τερηδόνα | Έλκος | Κοιλότητα | NO | Variance <5% |
| 20 | Sealant | Sealant | Drill | Cover | Κάλυψη Οπών και Σχισμών | Τροχίζω | Καλύπτω | **YES** |  |
| 21 | Periodontal | Periodontal | Gums | Palate | Περιοδοντικό | Ούλα | Υπερώα | **YES** |  |
| 22 | Analgesia | Analgesia | Vitamin | Aspirin | Αναλγησία | Βιταμίνη | Ασπιρίνη | **YES** |  |
| 23 | Fistula | Fistula | Wart | Draining | Συρίγγιο | Εξόγκωμα | Παροχέτευση | **YES** |  |
| 24 | Hyperemia | Hyperemia | Saliva | Blood | Υπεραιμία | Σάλιο | Αίμα | **YES** |  |
| 25 | Abscess | Abscess | Mucus | Pus | Απόστημα | Πύον | Βλέννα | NO | Variance <5% |
| 26 | Bruxism | Bruxism | Swallow | Grind | Βρυγμός | Καταπίνω | Τρίζω | **YES** |  |
| 27 | Hypoplasia | Hypoplasia | Defect | Intact | Υποπλασία | Ελαττωματικό | Ανέπαφο | NO | ITC <0.40 |
| 28 | Cellulitis | Cellulitis | Infection | Bleeding | Κυτταρίτις | Μόλυνση | Αιμορραγία | NO | ITC <0.40 |
| 29 | Temporomandibular | Temporomandibular | Joint | Neck | Κροταφογναθική | Άρθρωση | Λαιμός | **YES** |  |
| 30 | Apicoectomy | Apicoectomy | Root | Crown | Ακρορριζεκτομή | Ρίζα | Μύλη | **YES** |  |
| 31 | Filling | N/A |  |  | Έμφραξη | Δόντια | Ούλα | **YES** |  |
| 32 | Cavity | N/A |  |  | Κοιλότητα | Τερηδόνα | Ουλίτιδα | **YES** |  |
| 33 | Mouthrinse | N/A |  |  | Στοματικό Διάλυμα | Ξεπλένω | Καταπίνω | NO | Non-variant |
| 34 | Eruption | N/A |  |  | Ανατολή | Εμφάνιση | Απώλεια | **YES** |  |
| 35 | Panoramic | N/A |  |  | Πανοραμική | Ακτινογραφία | Κλινική | NO | TD p>0.05 |
| 36 | Plaque | N/A |  |  | Πλάκα | Μαλακό | Σκληρό | NO | ITC <0.40 |
| 37 | Veneer | N/A |  |  | Στεφάνη | Θήκη | Σφράγισμα | NO | TD p>0.05 |
| 38 | Fracture | N/A |  |  | Κάταγμα | Δόντι | Ούλο | NO | ITC <0.40 |
| 39 | Diagnosis | N/A |  |  | Διάγνωση | Συμπέρασμα | Επιλογή | NO | Non-variant |
| 40 | Incisor | N/A |  |  | Τομέας | Μπροστά | Πίσω | YES |  |
| 41 | Splint | N/A |  |  | Ακινητοποίηση | Σταθερό | Κινητό | YES |  |
| 42 | Mouth guard | N/A |  |  | Νάρθηκας | Δόντια | Χείλη | YES |  |
| 43 | Avulsion | N/A |  |  | Εκγόμφωση | Απώλεια | Σπάσιμο | YES |  |
| 44 | Ankylosis | N/A |  |  | Αγκύλωση | Οστό | Χείλος | NO | ITC <0.40 |

**Supplemental Table**. REALD-99, REALD-30, OHLA-E, GROHL pool, and (final) GROHL-20 items list, in English and Greek language.
